# Immediate consecutive microvascular decompression for bilateral classical trigeminal neuralgia

**DOI:** 10.3389/fneur.2024.1496656

**Published:** 2024-11-26

**Authors:** Mauro Alberto Segura-Lozano, Mario Alexis del Real-Gallegos, Pedro Mendoza-Lemus, Octavio Carranza-Rentería, Yael Rodrigo Torres-Torres, Alejandro González-Silva, Arturo Santoyo-Pantoja, Aarón Giovanni Munguía-Rodríguez

**Affiliations:** Neurología Segura Medical Center, Hospital Angeles Morelia, Morelia, Mexico

**Keywords:** bilateral trigeminal neuralgia, microvascular decompression, neuropathy, facial pain, neurovascular conflict

## Abstract

**Background:**

Classical trigeminal neuralgia (TN) is characterized by sudden, severe facial pain, typically resulting from a neurovascular conflict affecting the trigeminal nerve. In rare cases, both nerves are affected simultaneously causing bilateral TN (BTN), increasing the complexity of the treatment. Microvascular decompression (MVD) is a well-established treatment for TN; however, the experience with immediate consecutive bilateral MVD procedures is limited and requires further evaluation.

**Objective:**

To evaluate the safety and efficacy of immediate consecutive bilateral MVD in patients with severe BTN compared to non-consecutive bilateral MVD procedures.

**Methods:**

A retrospective analysis was conducted on 15 patients with BTN who underwent bilateral MVD. The data on clinical presentation, surgical technique, perioperative findings, complications, and follow-up outcomes of three cases of BTN treated with consecutive bilateral MVD surgeries were analyzed and compared to 12 who received separated procedures. Moreover, a detailed presentation of the three cases of consecutive MVD is provided to illustrate clinical decision-making, surgical nuances, and individual outcomes.

**Results:**

Both groups achieved significant pain relief (*p* < 0.001) without notable differences in Barrow Neurological Institute (BNI) pain intensity score (*p* = 0.305), indicating that both approaches were equally effective. The consecutive MVD group experienced a shorter total surgical duration (*p* = 0.025), while postoperative complications were comparable (*p* = 0.077), mostly transient with no major adverse events or mortality. At the last follow-up, the patients remained pain-free without recurrence of TN symptoms.

**Conclusion:**

Consecutive bilateral MVD is a safe and effective option, comparable to non-consecutive procedures for treating BTN. This approach provides a viable alternative for patients with severe bilateral symptoms or when medical constraints limit the possibility of two separate surgeries. Further studies with larger cohorts and extended follow-up periods are needed to support these results.

## 1 Introduction

Trigeminal neuralgia (TN) is a syndrome characterized by sudden, paroxysmal episodes of severe facial pain involving one or more branches of the trigeminal nerve (cranial nerve V, CN V) (1). This facial pain is often triggered by innocuous stimuli, causing significant discomfort and functional impairment in daily activities (2). The pathogenesis of classical TN is described as a neurovascular conflict (NVC) between the CN V and adjacent blood vessels (1). Generally, TN affect one side of the face and rarely occurs in both sides. Bilateral TN (BTN) is rare in clinical practice, with an incidence ranging from 0.6 to 5.9% of all TN cases (3).

Treatment options for BTN comprise those for unilateral TN (UTN) and include pharmacotherapy, percutaneous destructive procedures, gamma-knife radiosurgery, and microvascular decompression (MVD) (2). A thorough evaluation of each patient’s specific characteristics is necessary to select the most appropriate treatment. The MVD surgery has been shown to provide superior long-term outcomes with minimal complications and low morbidity and mortality rates compared to ablative procedures (1, 2, 4). The aim of MVD is to relieve pressure on the nerve by carefully separating the affected CN V from the compressing blood vessels, and strategically placing an autologous or artificial insulating material, thereby reducing or eliminating the pain associated with TN (5–7).

In cases of classical BTN, two MVD surgeries are typically performed with a recovery period between them (8, 9). However, in some instances, the severity of bilateral pain may require performing the second surgery without delay. This article presents three cases of patients with BTN who underwent MVD via two immediate consecutive bilateral craniotomies and compares the clinical data and outcomes with those of patients who received two separate MVD procedures for BTN.

## 2 Materials and methods

### 2.1 Study design

This retrospective, single-center study aimed to evaluate the safety and efficacy of immediate consecutive MVD for classical BTN. For comparison, clinical data and outcomes were analyzed against non-consecutive MVD procedures. Data were collected from clinical and surgical records, and statistical analyses were performed to identify differences between the two surgical approaches.

### 2.2 Inclusion criteria

From January 2011 to July 2024, a total of 2,166 patients with classical TN were treated at our clinic, with 143 (6.6%) presenting with BTN. During this period, we performed 85 MVD surgeries on 70 patients with BTN. Among these, 15 patients underwent bilateral MVD, including three who received consecutive surgeries. These three patients were selected for consecutive procedures due to the severity of their bilateral symptoms and resistance to pharmacological or other surgical treatments. Informed consent was obtained from all three patients, and the surgical interventions received approval from the institutional review board prior to the procedures.

### 2.3 Preoperative procedure

Each patient underwent a clinical evaluation by a multidisciplinary team including neurosurgeons, internists, and anesthesiologists to assess their suitability for surgery. Preoperative assessments included a comprehensive neurological examination, laboratory tests (lipid profile, thyroid function, glucose levels, etc.), and an evaluation of comorbidities and overall health status. Additionally, an imaging 3D-FIESTA MRI was performed to identify vascular contact with the cisternal portions of both CN V. The imaging data were used to plan the surgical approach and anticipate potential complications, such as prominent bony structures.

### 2.4 Surgical procedures

Patients were positioned in the lateral decubitus position with a 45° head rotation to allow optimal access to the cerebellopontine angle. The surgical intervention commenced with a retrosigmoid craniotomy on the side with the lower potential risk of complications, as determined by preoperative imaging and clinical evaluation. The surgical approach involved careful exploration of the cerebellopontine angle to identify and separate vascular structures in contact with the CN V. Teflon pads were meticulously placed to insulate the nerve from compressing vessels, while any conflicting veins were coagulated and resected when necessary.

Once decompression was achieved on the first side, the craniotomy was closed, and the patient was repositioned to allow access to the contralateral side. A second retrosigmoid craniotomy was then performed, employing the same technique. In cases where a prominent suprameatal tubercle obstructed visualization, bone drilling was performed to enhance the microsurgical view. Throughout the procedures, total intravenous anesthesia was maintained.

### 2.5 Postoperative evaluation

Postoperatively, 3D reconstructed CT scans were obtained to confirm the correct placement of the Teflon pads and to identify potential complications, such as pneumocephalus. Patients were closely monitored for adverse effects, and clinical follow-up was conducted for a minimum of 6 months. In addition, a long-term follow-up plan has been established, with evaluations every 6 months for a minimum of 3 years to assess sustained pain relief, detect potential recurrence, and monitor for late complications.

### 2.6 Statistical analysis

A statistical analysis was conducted to compare clinical and surgical data between consecutive and non-consecutive MVD surgeries for BTN. Independent samples t-tests or Mann–Whitney *U* tests were employed to compare variables such as age, surgical duration, and complication rates, depending on normality assessed by the Shapiro–Wilk test. Differences in Barrow Neurological Institute (BNI) pain intensity scores and symptom duration between groups were analyzed using the Mann–Whitney *U* test, while pre- and postoperative changes in BNI scores within groups were evaluated using the Wilcoxon signed-rank test. Statistical significance was set at *p* < 0.05. All analyses were performed using SPSS Version 25 (IBM® SPSS® Statistics, Chicago, IL, United States).

## 3 Results

### 3.1 Case 1

A 60-year-old Mexican woman presented to our clinic with a 20-year history of episodic, electric shock-like pain in the right V1, V2, and V3 branches of the CN V, and similar left facial pain in the V2 and V3 branches for the past 2 years. Initially, the patient sought dental care, which led to the extraction of four molars. Subsequently, she was treated by neurologists who diagnosed her with BTN and managed her condition with pharmacotherapy, including 1,200 mg/day of carbamazepine and 50 mg/day of dexketoprofen. However, due to the long history of pharmacological treatments and the current high doses of medication, the patient reported persistent side effects, including nausea, dizziness, and vomiting. She had no history of prior surgical interventions.

**Figure 1:**
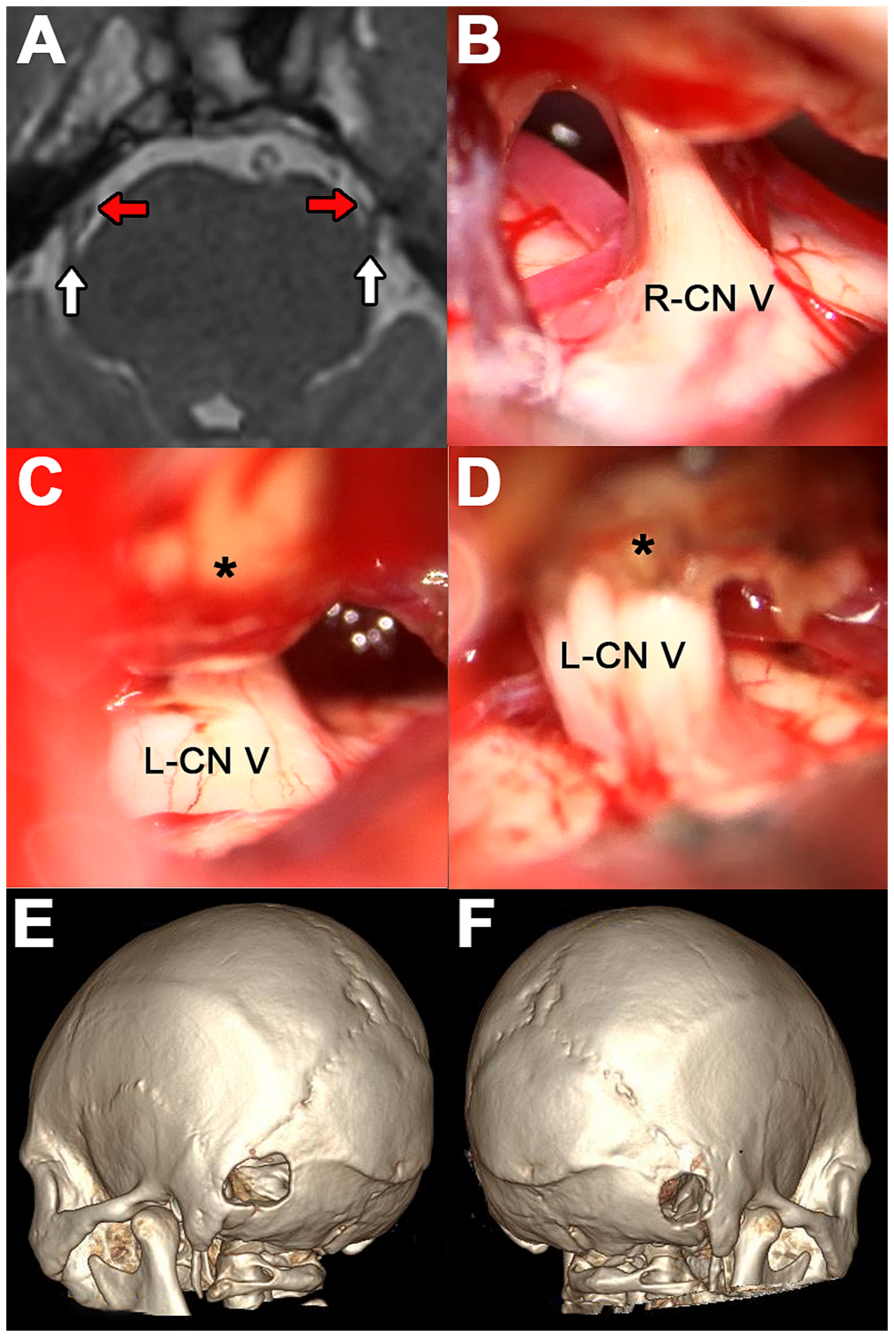
Case 1. (A) Axial 3D-FIESTA MRI at the level of the pons. The white arrows indicate the cisternal portion of CN V and the red arrows the contact vessels. (B) Surgical exploration on the right side showing SCA as the culprit vessel. (C) Suprameatal tubercle (asterisk) obstructing visualization of the left CN V. (D) Left-sided surgical exploration showing drilled suprameatal tubercle (asterisk) and redundant SCA as the offending vessel. (E,F) 3D reconstruction CT scan showing left and right retrosigmoid craniotomy, respectively.

During the clinical examination, severe bilateral facial pain was triggered by touch and eating. Her body mass index (BMI) was 26. A 3D-FIESTA MRI demonstrated vascular contact over the cisternal portion of both CN V (Figure 1A). Laboratory tests revealed slightly elevated cholesterol and triglyceride levels, along with elevated TSH and reduced free T4 and T3. The patient indicated being under treatment with 20 mg/d of atorvastatin and 50 mcg/d of levothyroxine to manage her comorbidities. Four months after the initial consultation, the patient’s bilateral pain had become disabling, severely affecting her ability to swallow and eat. Given the worsening condition, intolerable medication side effects, and the patient’s financial limitations, two consecutive MVD procedures were suggested. After discussing the risks and benefits, bilateral surgery was approved by the patient, her family, and authorized by the hospital’s ethics committee. The patient’s physical health was assessed by our team of internists and anesthesiologists, who evaluated her potential response to anesthesia and the risk of cardiac and respiratory complications. Following a comprehensive evaluation, it was concluded that she was in suitable condition to undergo both consecutive procedures.

Initially, a right MVD was performed. During the procedure, the superior cerebellar artery (SCA) and an unnamed vein were identified compressing the CN V (Figure 1B). The artery was gently separated from the nerve, and a Teflon pad was interposed. The vein was coagulated and resected. Immediately after closing the right approach, the patient was repositioned to expose the left side for a second, consecutive retrosigmoid approach. Upon opening the dura, a suprameatal tubercle approximately 3 mm in size obstructed the surgical view (Figure 1C). Drilling of this bony structure was necessary until a redundant SCA and another unnamed vein could be identified and separated using Teflon felts (Figure 1D). The suprameatal tubercle was not detected on the presurgical MRI (Figure 1A).

Postoperatively, a CT scan with 3D reconstruction was performed (Figures 1E,F). The patient reported hypoesthesia on the right side and paresthesia, otic fullness, and tinnitus on the left side. All these adverse events were transient. There were no major complications. Pain relief was immediate on both sides, with continued satisfactory results at 6 months of follow-up.

### 3.2 Case 2

A 58-year-old Chilean woman presented with an eight-year history of TN on the left side, involving only the V2 division. Over the past 3 years, she developed pain in the V2-V3 distribution on the contralateral side. The patient initially consulted a dentist who extracted a molar and was subsequently seen by a neurologist who diagnosed her with BTN. The patient reported an allergy to carbamazepine and was instead prescribed pregabalin and amitriptyline. These medications controlled her pain for the past 2 years with progressively increasing doses. Recently, she experienced severe pain crises, which were managed with tramadol. Two years ago, she underwent a left-sided radiofrequency thermocoagulation which only controlled the pain for 3 months.

**Figure 2:**
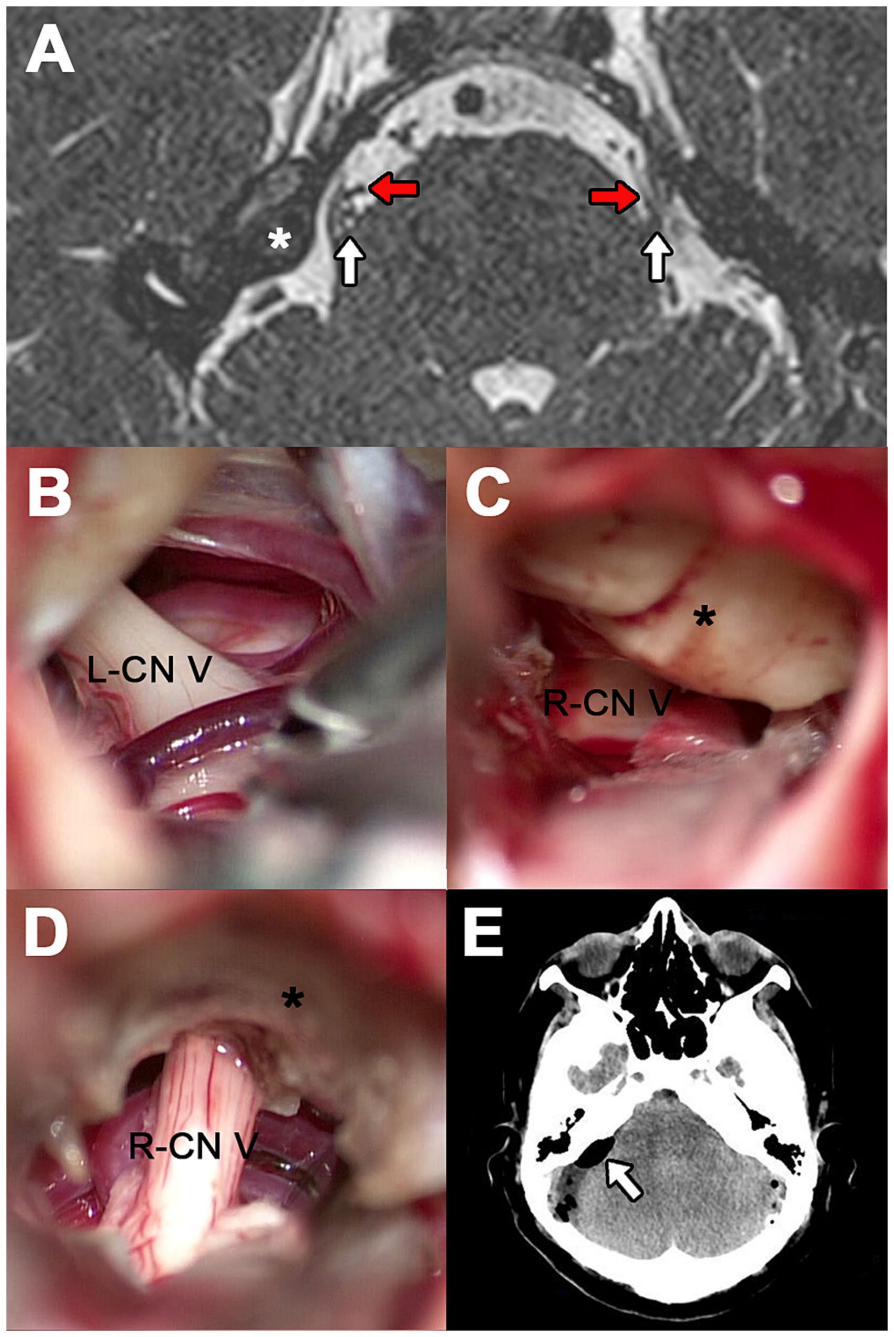
Case 2. (A) 3D-FIESTA MRI showing both CN V at the level of the pons (white arrows). The red arrows indicate the contact vessels and the asterisk shows a lobulated osseous prominence of the right petrosal surface. (B) SCA and a circumflex vein over the left CN V. (C) Prominent suprameatal tubercle (asterisk) obstructing the clear visualization of the CN V. (D) Redundant AICA compressing the CN V revealed after drilling the suprameatal tubercle (asterisk). (E) Postoperative cranial CT scan. The arrow indicates the pneumocephalus in the right anterolateral cistern of the brainstem.

During clinical examination, severe bilateral pain was triggered by touch and exposure to cold and wind, with more frequent pain episodes on the right side. Her BMI was 24. Laboratory tests showed no remarkable abnormalities. However, her blood pressure was elevated (151/93 mmHg), and she reported being under treatment with losartan 50 mg/d. Furthermore, the Beck Depression Inventory (BDI-2) revealed moderate depression. A 3D FIESTA MRI revealed vascular contact over the cisternal portion of both CN V and a > 6 mm prominence of the petrosal surface of the right temporal bone was also identified (Figure 2A). The patient was prescribed pregabalin 150 mg/d and duloxetine 60 mg/d.

Given the MRI finding on the right side, which could complicate or prolong the surgery, it was decided to explore the left side first to ensure at least one successful MVD. During the initial procedure, the SCA and a circumflex vein were separated from the nerve by interposing Teflon felts (Figure 2B). Upon contralateral exploration, a large suprameatal tubercle exceeding 6 mm was encountered, obstructing the microsurgical corridor to the cisternal segment of the CN V. This required the drilling of the bony structure, which extended the surgical time by 75 min more than expected (Figure 2C). When the neurovascular conflict could be identified, the CN V was being contacted by a redundant AICA and SCA (Figure 2D). These vessels were gently transposed, and two Teflon pads were placed to insulate the nerve.

During the postoperative evaluation, the patient reported persistent headache, vertigo, right-sided hypoesthesia and left-sided tinnitus. A postoperative CT scan revealed a mild pneumocephalus located at the right anterolateral cistern of the brainstem, likely resulting from the extended drilling of the suprameatal tubercle (Figure 2E). The pneumocephalus was managed with 72 h of strict supine rest, hyperhydration and supplemental oxygen. Bilateral pain relief was achieved following the surgery, and the patient remains pain-free during the 6 months of follow-up. All complications were transient, and her emotional state has improved since the intervention.

### 3.3 Case 3

A 54-year-old Mexican woman was admitted to our clinic after experiencing seven consecutive days of severe pain crises that required hospitalization. She reported a two-year history of neuropathic pain characterized by electrical and burning sensations affecting the left V1, V2, V3, and right V3 branches of CN V. Bilateral pain began 3 years prior, with the onset occurring almost simultaneously, differing by only a few weeks. Initially, she consulted a dentist who extracted three molars. Her treatment regimen, prescribed by a neurologist and an algologist, included oxcarbazepine 1800 mg/d, pregabalin 300 mg/d, duloxetine 60 mg mg/d and tapentadol 100 mg/d. No previous surgical interventions were reported.

**Figure 3:**
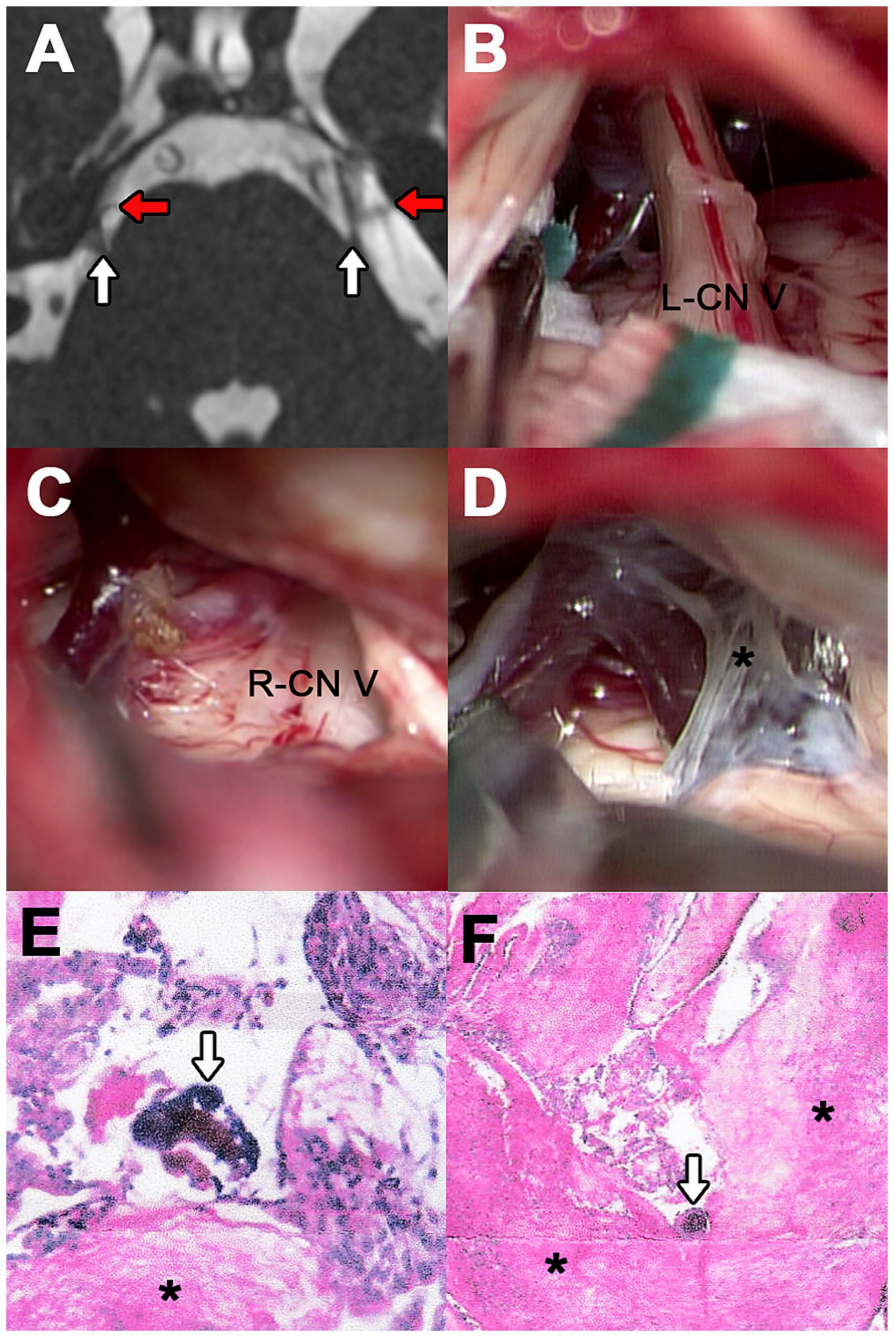
Case 3. (A) 3D-FIESTA MRI demonstrating both CN V at the level of the pons (white arrows). The red arrows point to the vessels making contact. (B) Surgical exploration on the left side showing SCA and a pontine vein. (C) Right neurovascular conflict caused by SCA and a vein tributary to the SPVC. (D) Right findings of altered arachnoid (asterisk) with a viscous consistency during the examination. (E,F) Results of hematoxylin and eosin staining of left and right arachnoid samples, respectively. The asterisk shows the area with fibrosis and the arrow indicates macrocalcifications.

During the clinical examination, severe bilateral pain was triggered by any contact with the face and presented with the same intensity on both sides. Her BMI was calculated to be 22. Laboratory tests indicated slightly elevated glucose, cholesterol, and triglyceride levels. The patient was managing type 2 diabetes mellitus and dyslipidemia with metformin 850 mg/d and atorvastatin 10 mg/d, respectively. A 3D-FIESTA MRI revealed vascular contact with the cisternal portions of both CN V (Figure 3A).

Two consecutive craniectomies were conducted to decompress the left and then the right CN V. On the left side, the neurovascular conflict involved the SCA and a pontine vein (Figure 3B). On the right side, the offending vessels were the SCA and a tributary vein to the superior petrosal vein complex (SPVC; Figure 3C). The arteries were separated from the nerve, and Teflon pads were interposed. The vein was coagulated and resected. Both explorations revealed severe thickening of the arachnoid membranes with a viscous consistency, which was meticulously excised, increasing the difficulty of the microsurgery (Figure 3D). The altered arachnoid tissue was stained with hematoxylin and eosin (H&E), which showed evidence of fibrosis and microcalcifications (Figures 3E,F).

Postoperatively, the patient reported tinnitus on the right side, while the left side exhibited grade II facial paralysis, ear fullness, and hypoesthesia. All complications were transient, with no major complications occurring. At the four-month follow-up, pain had completely resolved on the right side. On the left side, the patient reported occasional, less intense pain that did not require medication.

### 3.4 Comparison between consecutive and non-consecutive bilateral MVD for classical BTN

To further evaluate the effectiveness and safety of immediate consecutive MVD, we compared its outcomes with those of patients who underwent two non-consecutive MVD surgeries for classical BTN at our center (*n* = 12; Table 1).

**Table 1:** Characteristics of patients who underwent non-consecutive (1–12) and consecutive (A-C) MVD surgeries for BTN.

Case	Sex	Age at surgery		Years of symptoms /Affected side		Years between surgeries	Pre-surgical BNI score		Duration of surgery (min)		Surgical findings		Number of postoperative complications		Post-surgical BNI score	
		1st surgery	2nd surgery	1st surgery	2nd surgery		1st surgery	2nd surgery	1st surgery	2nd surgery	1st surgery	2nd surgery	1st surgery	2nd surgery	1st surgery	2nd surgery
1	F	54	58	7/L	2/R	4	V	V	161	144	ST, A	V, A	1	1	I	II
2	F	62	63	8/L	0.3/R	0.5	V	IV	137	153	SCA, V, A	SCA, V, A	1	2	I	I
3	F	34	37	2/R	0.25/L	2.6	IV	IV	166	193	V, A	SCA, V, A	1	3	I	I
4	F	71	73	33/L	15/R	2.5	V	V	199	224	SCA	SCA, V	2	2	I	I
5	F	55	55	10/L	15/R	0.4	V	V	145	156	SCA, V, A	SCA, A	2	1	I	I
6	F	26	30	2/L	1/R	3.6	V	IV	174	178	SCA, V, A	SCA, ST	2	1	II	II
7	F	76	81	15/L	7/R	4.7	V	V	206	118	V	SCA, V, A	1	1	I	I
8	M	43	46	2/L	0.5/R	3.3	IV	IV	182	158	SCA, AICA, V, A	SCA, V, A	3	2	I	I
9	F	51	51	10/L	9/R	0.3	V	V	136	150	V, A	SCA, A	3	2	II	II
10	F	41	45	4/R	0.5/L	3.6	IV	V	146	203	AICA, V, A	SCA, A	1	1	I	I
11	F	61	64	20/R	0.6/L	3.4	V	IV	235	220	SCA, V, A	SCA, V, A	3	2	III	II
12	F	60	66	23/R	0.5/L	6	V	V	184	152	SCA, V, A	SCA, A	1	2	II	III
A	F	60		20/R	2/L	0	V	V	295		SCA, V, A	SCA, V, A, ST	1	**3**	**I**	**I**
B	F	58		8/L	3/R	0	V	V	315		AICA, V	SCA, AICA, ST	2	3	I	I
C	F	54		3/L	3/R	0	V	V	280		SCA, V, A	SCA, V	3	1	I	II

F, Female; M, Male; L, Left; R, Right; SCA, Superior Cerebellar Artery; AICA, Anterior Inferior Cerebellar Artery; V, Vein; A, Altered Arachnoid; ST, Suprameatal Tubercle; BNI, Barrow Neurological Institute pain intensity score: I (no pain, no medication required), II (occasional pain, not requiring medication), III (persistent pain, adequately controlled with medication), IV (some pain, not adequately controlled with medication), V (severe pain/no pain relief).

The mean age of patients who underwent consecutive bilateral MVD was 57.7 years, while the mean ages for patients who had two separate MVD procedures were 52.8 years for the first procedure and 55.8 years for the second procedure. No significant differences in age were observed between patients undergoing consecutive MVD and those undergoing the first (*p* = 0.32) or second (*p* = 0.69) non-consecutive surgeries. The median duration of TN symptoms on the first operated side was 10 years for patients in the consecutive group and 11.7 years for those in the non-consecutive group (*p* = 0.292). Prior to the surgery of the second operated side, the duration of pain was 4.3 years for patients in the consecutive group and 2.3 years in the non-consecutive group (*p* = 0.268). Notably, contralateral pain developed in 66.6% of patients after their initial MVD with an average onset of 2.7 years. Additionally, in patients with non-consecutive surgeries, the average interval between the two procedures was 2.9 years. The mean operative time for consecutive MVD was 296.7 min, while the mean of combined operative time for both procedures in the non-consecutive group was 343.3 min, indicating a shorter total surgical time for consecutive procedures (*p* = 0.025).

Postoperative complications were assessed before discharge. The median number of complications was comparable between groups, with an average of 4.3 in the consecutive group and 3.3 in the non-consecutive group (*p* = 0.077). At 6 months the after surgery, pain outcomes of all patents could be evaluated using the BNI pain intensity scale (10). Both groups demonstrated significant improvement in BNI scores after surgery (*p* < 0.001). However, there was no significant difference in pain relief between the consecutive and non-consecutive MVD groups (*p* = 0.305), suggesting that both approaches can be equally effective in controlling pain in patients with BTN.

## 4 Discussion

Classical BTN is characterized by paroxysmal, electric shock-like pain on both sides of the face, typically affecting areas innervated by the CN V. The neurovascular conflict hypothesis is widely accepted for explaining the pathogenesis of classical TN. According to this theory, chronic compression of the CN V by abnormal blood vessel trajectories at the root entry zone (REZ) leads to inflammation and demyelination of the nerve root. This compression results in a “short circuit” of membrane potential, causing neuropathic pain triggered by innocuous stimuli (11, 12). Additionally, it has been described that patients with BTN have smaller posterior cranial fossae and cerebellopontine cistern volumes compared to healthy controls. This reduced volume is associated with an overcrowded posterior fossa, leading to a higher incidence of neurovascular conflict that ultimately may induce BTN (12).

The diagnosis of both BTN and UTN primarily relies on patient history and is supported by appropriate imaging studies (13). Clinical and imaging evaluations are essential not only for diagnosing TN but also for excluding secondary causes, such as tumors or multiple sclerosis (MS). The incidence of BTN accounts for 0.6 to 5.9% of all TN cases (3, 8). At our center, BTN has an incidence of 6.6% among cases of classic TN; however, this incidence rises to 8.7% when secondary and idiopathic TN cases are considered. Bilaterality is significantly more common in patients with a familial history of TN compared to those with UTN, suggesting a potential predisposition to symptomatic neurovascular compression in certain individuals and families (8, 14). Additionally, MS is associated with a higher incidence of BTN, with reports indicating that 4–10% of BTN patients also present MS (15, 16). However, none of the patients in our study showed evidence of MS during clinical examination, nor were they aware of any family history of TN.

Pharmacological treatment is the first-line option for managing both UTN and BTN. However, when medications fail to provide long-term pain control or cause intolerable side effects, surgical intervention should be considered. Various surgical approaches have been proposed for the treatment of these conditions, including ablative procedures, which offer quick pain relief but have limited long-term effectiveness (2, 17). MVD is another surgical technique to treat compressive neuropathies that directly addresses the underlying cause of symptoms. As a non-ablative surgery, MVD can preserve CN V function, thereby increasing pain control rates, and decreasing the risks of complications, recurrence rates, and the need for repeated interventions compared to ablative surgeries (3, 12, 17, 18). The success rate of MVD for UTN at 1-year follow-up is high (80–90%) (9), and is generally recommended as the primary surgical option in cases of classical TN when the patient’s overall condition is favorable. In contrast, ablative approaches are considered for atypical cases of TN, when there is an absence of neurovascular compression, when the patient faces increased perioperative risks, or when MVD has failed (19).

Similar to UTN, MVD can be a safe and effective treatment of BTN (12). However, the utility of performing separated MVD for primary BTN has been evaluated in only three studies, all of which were case series (8, 9, 14). In Pollack’s study involving 35 BTN patients over 14 years, good or excellent results were achieved on 89% of the treated sides, and 74% maintained good or excellent pain relief during a mean follow-up of 75 months (8). Tacconi and Miles reported poorer responses in 16 BTN cases treated over 14 years, requiring further medical management and additional ablative procedures, possibly due to including patients with idiopathic BTN and TN associated with MS or Charcot–Marie–Tooth disease (14). Zhao et al. described 13 BTN cases treated with MVD over a two-year period, achieving good or excellent symptom control on 92.3% of the treated sides; among these, nine patients underwent contralateral MVD within 1 year with excellent outcomes (9). In BTN patients, contralateral surgery is recommended at least 3 months after the initial MVD (12). However, Tun et al. reported performing two separate MVD procedures on an older BTN patient with a two-week interval, the shortest period reported at the same institution. The patient did not experience any complications during follow-up (17).

Only two previous reports mention having performed bilateral MVD consecutively (8, 14). However, neither provided detailed information on the intervention, perioperative management or follow-up. It is mentioned that consecutive bilateral MVD is a feasible and reasonable option when the MRI shows bilateral contact, and the pain on both sides is equally severe, such that treating only one side would not reduce the medication or improve the patient’s quality of life (14). Recently, a report described the possibility of decompression on both sides through a single unilateral approach. The authors performed bilateral MVD using a unilateral craniectomy in two patients with an enlarged superior trigeminal nerve space, successfully identifying and releasing both neurovascular conflicts. However, they indicated that not every BTN patient could be treated this way due to anatomical limitations (3).

Based on our experience, a thorough preoperative physiological evaluation should precede consideration of a double procedure. Ensuring the absence of significant metabolic conditions or comorbidities that could complicate surgery is crucial. Patients should ideally undergo this approach at their optimal weight to minimize anesthetic and obesity-related risks. Our three patients were relatively young and healthy, with manageable comorbidities and no significant contraindications to surgery. Additionally, other palliative surgical and non-surgical options were discussed with the patients. The decision for surgery was made jointly by the patient, their family, the hospital ethics committee, and an expert medical team, who evaluated the patient’s health status and potential surgical risks. On the other hand, it is also important to consider the patient’s economic situation. By performing two consecutive interventions, the patient undergoes a single process for anesthesia, hospitalization, transportation, and recovery, thereby reducing overall costs.

Bilateral pain typically occurs asynchronously, with one side often more symptomatic than the other. Generally, the side with the most severe pain is treated surgically first (12). However, after treating the more symptomatic side, contralateral symptoms often become more disabling over time, necessitating continued high-dose medication (8). In such cases, patients might benefit from an immediate consecutive MVD. As with unilateral MVD, a criterion for surgery is the confirmation of vascular contact on the nerve via MRI (9). Imaging studies can also help identify findings that may increase surgical complexity. In the second case, a prominent right suprameatal tubercle was identified on the preoperative MRI, prompting us to operate on the opposite side first. While it is generally recommended to address the more symptomatic side first, we suggest prioritizing the side with fewer risks and requires shorter surgical time. This strategy aims to ensure at least one successful MVD and provides additional time to address the more complex side. Operating on the more complicated side first could result in the second surgery being postponed due to complications or unforeseen findings. In this instance, drilling of the suprameatal tubercle extended the surgical time and resulted in a pneumatocele, underscoring the importance of minimizing intradural exposure and avoiding prolonged CSF drainage during microsurgery. In the third case, thickened arachnoid tissue was observed on both sides, complicating vessel and nerve manipulation. This altered tissue might contribute to neurovascular conflict by forming adhesions with adjacent structures, necessitating its careful removal. The etiology of this alteration remains unclear; however, the presence of fibrosis and microcalcifications suggests the involvement of a chronic inflammatory process (20).

Some authors are reluctant to perform an early second MVD due to reports of contralateral symptom resolution following unilateral MVD. It has been suggested that bilateral symptom relief may occur when the contralateral vessel is displaced during the removal of the ipsilateral offender or when CSF release during surgery leads to separation of neurovascular contact (9). However, there are also reports of early and late iatrogenic contralateral TN after MVD for unilateral TN (21, 22). Achieving bilateral pain relief in a single surgical session can benefit patients by avoiding two separate procedures with their associated risks and complications, reducing patient costs, preventing two recovery periods, and avoiding prolonged unnecessary pain. While the aim of this study is to support the safety and efficacy of consecutive MVD, the small sample size and short follow-up period limit the generalizability of the findings. Nonetheless, this study provides a foundation for further research. Increasing the cohort size and extending the follow-up period in future studies will be essential to confirming the effectiveness and safety of this approach in patients with severe bilateral pain who meet the optimal criteria, such as good overall health, appropriate weight, and a diagnosis of classic BTN.

## 5 Conclusion

The cases presented herein demonstrate the feasibility and effectiveness of performing two immediate consecutive MVD surgeries for the treatment of synchronous classical BTN. Our results support that, when patients are appropriately selected based on their clinical profile and imaging studies, this approach can provide significant pain relief with minimal complications in a specialized center. This approach may be particularly viable for patients with severe bilateral symptoms or other constraints that prevent two separate surgeries. Nevertheless, larger case series and extended follow-up studies are necessary to further support the safety and long-term efficacy of this surgical strategy.

## Data Availability

The datasets presented in this article are not readily available because of ethical and privacy restrictions. Requests to access the datasets should be directed to the corresponding author.
